# Supplementary material for: Feasibility and acceptability of integrating a multicomponent breastfeeding promotion intervention into routine health services in private health facilities in Lagos State, Nigeria: A mixed methods process evaluation
Source: PLoS One. 2024 Apr 26;19(4):e0301695. doi: 10.1371/journal.pone.0301695 (PMC11051595; doi:10.1371/journal.pone.0301695)
Supplement: S1 File — (DOCX) [file pone.0301695.s001.docx]

**S1 File. In-depth Interview guide**

Providers should be individuals trained by A&T for this study. Mangers/owners and providers must be 18 years or older.

As you know, for the last year, your health facility has been involved in a project with Alive & Thrive and EHAI to improve breastfeeding counseling to pregnant and breastfeeding women in private health facilities in Lagos.

1. First, please tell me how the Alive & Thrive/EHAI infant and young child feeding project was implemented in your facility. What happened first and then next and so on?
2. In what ways did you participate in the Alive & Thrive / EHAI project?
3. Overall, what were your experiences with the Alive & Thrive/EHAI project?
4. Overall, what were the factors that helped you to integrate breastfeeding counseling and support into the services at your facility during this project?
5. Overall, what were the main barriers to integrating breastfeeding counseling and support into the services at your facility during this project?
6. How does the provision of breastfeeding counseling and support affect service delivery in your facility? **Probe** on staff time, staff availability, cost
7. What motivated you to participate in this project and include breastfeeding counseling and support as part of maternal and child health services at your facility?
8. What did you think about the training Alive & Thrive and EHAI provided to staff in this facility? **Probe** on frequency of training, duration of training, topics covered.

What topics were covered during the training? **Probe** anything else?

Which topics did you think were most useful? Why?

Which topics did you think were least useful? Why?

*(Info for interviewers - these were the topics covered during the training: initiation of breastfeeding within the 1^st^ hour of birth, initiating skin-to-skin contact, positioning and attachment, expression of breastmilk, frequency of breastfeeding, breastfeeding challenges, the 10 steps to successful breastfeeding, 10 Key points in the BMS (Breast milk Substitute) code, enrollment for phone and WhatsApp messaging.)*

- 1. What were the benefits for you or your staff of receiving the training on breastfeeding?
  2. What challenges, if any, did you or your staff face related to the breastfeeding training?
  3. Did you or your staff participate in any refresher training conducted by Alive & Thrive and EHAI? **Probe** what topics were covered? What topics did you find most/least useful? What were the benefits or challenges (if any) related to the refresher training?

1. What types of health education materials did your facility receive as part of the Alive & Thrive/EHAI project?

*(Info for interviewers – Facilities were supposed to receive counseling manuals, posters on early initiation of breastfeeding/exclusive breastfeeding/10 points on code of marketing BMS, pamphlets on nutrition during pregnancy/breastfeeding and how to breastfeed, Z-cards, and flash drives with Start Strong IYCF messages)*

- 1. What did you think about the health education materials provided by Alive & Thrive and EHAI? **Probe** on adequacy of number of materials, quality of materials, training on how to use the materials.
  2. What were the benefits to you or your staff and clients of the health education materials provided?
  3. Which of the health education materials were most useful to you or your staff? Why?
  4. Which of the health education materials do you think were most useful to your clients? Why?
  5. What barriers, if any, did you or your staff and clients have with using the breastfeeding health education materials?
  6. Which of the health education materials were least useful to you or your staff? Why?
  7. Which of the health education materials do you think were least useful to your clients? Why?

1. Did your facility receive a cell phone as part of the Alive & Thrive and EHAI project? If so, how was the phone used? **Probe** on text messaging related to breastfeeding and WhatsApp groups related to breastfeeding.
   1. What benefits did the breastfeeding phone messaging provide to you or your staff?
   2. What were the benefits to your clients of receiving phone messages on breastfeeding?
   3. What barriers or challenges did you or your staff face related to sending phone messages on breastfeeding? **Probe** on issues with the phone itself, with getting messages out, with responding to queries from clients
   4. What were the barriers or challenges experienced by your clients related to phone messages on breastfeeding? **Probe** on issues with receiving the messages, understanding the messages, finding the messages to be unhelpful, finding the messages to be intrusive
   5. What benefits did the breastfeeding WhatsApp groups provide to you or your staff?
   6. What were the benefits to your clients of participating in breastfeeding WhatsApp groups?
   7. What were the barriers or challenges experienced by you or your staff related to the breastfeeding WhatsApp groups? **Probe** on issues with the phone itself, with getting messages out, with running the WhatsApp group, with responding to queries from group participants
   8. What were the barriers or challenges experienced by your clients related to the breastfeeding WhatsApp groups? Probe on issues with joining the group or participating in the group, understanding the content, finding the content to be unhelpful, finding the group to be intrusive
2. What were your experiences or the experiences of your staff in conducting group breastfeeding counseling at this facility? **Probe** on frequency of group breastfeeding counseling and who usually conducted it.
   1. What factors helped you or your staff to conduct group breastfeeding counseling in this facility?
   2. What were the challenges for you or your staff in conducting group breastfeeding counseling?
   3. How, if at all, did the group breastfeeding counseling at your facility change since the Alive & Thrive/EHAI project started?
   4. What are your clients’ opinions about group breastfeeding counseling at your facility since the Alive & Thrive/EHAI project started?
3. What were your experiences or the experiences of your staff in conducting one-on-one breastfeeding counseling at this facility? **Probe** on frequency of one-on-one breastfeeding counseling and who usually conducted it.
   1. What factors helped you or your staff to conduct one-on-one breastfeeding counseling?
   2. What were the challenges for you or your staff in conducting one-on-one breastfeeding counseling?
   3. How, if at all, did one-one-one breastfeeding counseling change at your facility since the Alive & Thrive/EHAI project started?
   4. What are your clients’ opinions of one-on-one breastfeeding counseling at your facility since the Alive & Thrive/EHAI project started?
4. Please tell me about your interactions with Alive & Thrive and EHAI project staff. How often did they come to your facility? In what ways did they support you to integrate breastfeeding counseling into the services at your facility?
   1. Did you or your staff engage with breastfeeding coaches, who were part of the Alive & Thrive/EHAI project? **Probe** How frequently did the breastfeeding coaches support you and/or other health workers at your facility? What did you like about interactions with breastfeeding coaches? What did you dislike about interactions with breastfeeding coaches?
   2. Did you or your staff participate in any monthly review meetings? **Probe** How often? What was discussed during these monthly review meetings? What did you like about these monthly review meetings? What did you dislike about these monthly review meetings?
   3. Did Alive & Thrive / EHAI project staff support you in using IYCF registers? In what way? **Probe** What did you like about the IYCF registers? What did you dislike about the IYCF registers?
   4. Did Alive & Thrive / EHAI project staff support you in using national data registers or NHMIS tools? In what way?
5. What are you plans for continuing breastfeeding-related activities in your clinic after the Alive & Thrive/EHAI project ends?
   1. Which activities will be easiest to continue? Why?
   2. Which activities will be difficult to continue? Why?

**Thank you for taking the time to answer these questions. Now I have just a few questions about you.**

1. How old are you?
2. How many children do you have?
3. Are you male or female? (Note to interviewer: We need this information on the recording so it becomes part of the transcript)
4. Are you the owner/manager or a health provider? (Note to interviewer: We need this information on the recording so it becomes part of the transcript)
5. How many years have you worked at or owned this clinic?
