# Supplementary material for: Feasibility and acceptability of integrating a multicomponent breastfeeding promotion intervention into routine health services in private health facilities in Lagos State, Nigeria: A mixed methods process evaluation
Source: PLoS One. 2024 Apr 26;19(4):e0301695. doi: 10.1371/journal.pone.0301695 (PMC11051595; doi:10.1371/journal.pone.0301695)
Supplement: S2 File — (DOCX) [file pone.0301695.s002.docx]

**S2 File. Client-Provider Interaction Observations Tool**

BEFORE OBSERVING THE VISIT, MAKE SURE THAT:

- YOU HAVE RECEIVED INFORMED CONSENT FROM BOTH THE PROVIDER AND THE CLIENT
- THE PROVIDER KNOWS THAT YOU ARE NOT THERE TO EVALUATE HIM OR HER
- THAT YOU ARE NOT AN EXPERT TO BE CONSULTED DURING THE SESSION
- REMIND THE PROVIDER AND CLIENT THAT THEY MAY ASK YOU TO LEAVE AT ANY TIME IF THEY FEEL UNCOMFORTABLE.

ASK THE WOMAN HER YOUNGEST CHILD’S AGE BEFORE THE VISIT BEGINS.

1. Before the visit begins, what is her current month of pregnancy OR the age in months of her youngest child?

Month of Pregnancy: _______ OR Child’s age in months: ____ ____

1. Time observation started ____ ____ : ____ _____
2. Date: Day ________ Month ________ Year ________
3. Name of observer: ________________________________________
4. Client Participant ID #: _____ _____ (USE SAME ID AS FOR SURVEYS)
5. Name of facility (circle one):

| Intervention private facility #1 | Comparison private facility #1 |
| --- | --- |
| Intervention private facility #2 | Comparison private facility #2 |
| Intervention private facility #3 | Comparison private facility #3 |
| Intervention private facility #4 | Comparison private facility #4 |
| Intervention private facility #5 | Comparison private facility #5 |
| Intervention private facility #6 | Comparison private facility #6 |
| Intervention private facility #7 | Comparison private facility #7 |
| Intervention private facility #8 | Comparison private facility #8 |
| Intervention private facility #9 | Comparison private facility #9 |
| Intervention private facility #10 | Comparison private facility #10 |

1. Types of providers observed 8. Sex of provider 9. Provider ID # 10. 1^st^ obs?

0=male, 1=female 0=no, 1=yes

A__________________________ _____ P ___ ___ _____

B__________________________ _____ P ___ ___ _____

C__________________________ _____ P ___ ___ _____

D__________________________ _____ P ___ ___ _____

E__________________________ _____ P ___ ___ _____

F__________________________ _____ P ___ ___ _____

G__________________________ _____ P ___ ___ _____

H__________________________ _____ P ___ ___ _____

I___________________________ _____ P ___ ___ _____

RECORD WHICH PROVIDER(S) DID ANY OF THE FOLLOWING AND IF THE PROVIDER DID NOT DO ANY OF THE FOLLOWING (1=yes, 0=no) | D=doctor, MN=midwife/nurse, AN=auxiliary nurse, CHEW=community health extension worker, HS=health staff, BC=breastfeeding champion

|  |  | **Type of health provider** | | | | | |
| --- | --- | --- | --- | --- | --- | --- | --- |
|  |  | D | MN | AN | CHEW | HS | BC |
|  | **Privacy/Confidentiality** |  |  |  |  |  |  |
| 11. | Ensured visual privacy |  |  |  |  |  |  |
| 12. | Ensured auditory privacy |  |  |  |  |  |  |
| 13. | Assured the client orally of confidentiality |  |  |  |  |  |  |
|  | **Advice on IYCF** |  |  |  |  |  |  |
|  | FOR PREGNANT WOMEN |  |  |  |  |  |  |
| 14. | Asked if the client intends to breastfeed |  |  |  |  |  |  |
| 15. | Encouraged breastfeeding |  |  |  |  |  |  |
| 16. | Discussed importance of early initiation of breastfeeding |  |  |  |  |  |  |
| 17. | Discussed importance of early skin-to-skin contact |  |  |  |  |  |  |
| 18. | Discussed essential newborn care (e.g., kangaroo care) |  |  |  |  |  |  |
| 19. | Discussed “rooming-in” with infant after birth |  |  |  |  |  |  |
| 20. | Explained colostrum and its benefits |  |  |  |  |  |  |
| 21. | Explained meaning of exclusive breastfeeding (i.e., no other foods or liquids, even water) |  |  |  |  |  |  |
| 22. | Discussed the benefits of exclusive breastfeeding |  |  |  |  |  |  |
| 23. | Advised to give glucose water before mother’s milk comes in |  |  |  |  |  |  |
| 24. | Advised to feed infant formula |  |  |  |  |  |  |
| 25. | Advised to feed infant other foods |  |  |  |  |  |  |
|  | FOR ALL CHILDREN 0-6 MONTHS |  |  |  |  |  |  |
| 26. | Asked if the client is breastfeeding |  |  |  |  |  |  |
| 27. | Asked if the client is giving infant formula |  |  |  |  |  |  |
| 28. | Advised to feed infant formula |  |  |  |  |  |  |
| 29. | Asked if the client is giving the infant water |  |  |  |  |  |  |
| 30. | Asked if the client is giving other fluids |  |  |  |  |  |  |
| 30. | Asked if the client is giving food |  |  |  |  |  |  |
| 31. | Explained meaning of exclusive breastfeeding (i.e., no other foods or liquids, even water) |  |  |  |  |  |  |
| 32. | Discussed the benefits of exclusive breastfeeding |  |  |  |  |  |  |
| 33. | Advised to feed water, other fluids, and/or food |  |  |  |  |  |  |
| 34. | Advised the client when to start complementary feeding |  |  |  |  |  |  |
| 35. | Asked if the client has any problem related to breastfeeding the child |  |  |  |  |  |  |
| 36. | Provided advice about and/or demonstrations to address breastfeeding problems |  |  |  |  |  |  |
| 37. | Advised to continue breastfeeding if mother or child is ill |  |  |  |  |  |  |
| 38. | Advised to feed expressed breast milk if mother is away from child |  |  |  |  |  |  |
| 39. | Discussed how to express and store breast milk |  |  |  |  |  |  |
| 40. | Advised not to give infant artificial teats, pacifiers or suckers |  |  |  |  |  |  |
| 41. | Encouraged breastfeeding on demand |  |  |  |  |  |  |
|  | **Additional Provider Actions** |  |  |  |  |  |  |
| 42. | Discussed when to come for the next visit |  |  |  |  |  |  |
| 43. | Wrote in client’s health record |  |  |  |  |  |  |
| 44. | Used any teaching tools and/or visual aids for counseling on IYCF |  |  |  |  |  |  |
| 45. | Provided client with take-home educational materials |  |  |  |  |  |  |
| 46. | Encouraged client to participate in peer support groups (e.g., WhatsApp group, in-person meetings) |  |  |  |  |  |  |
| 47. | Encouraged client’s husband to participate in WhatsApp group |  |  |  |  |  |  |
|  | **Quality of Interaction** |  |  |  |  |  |  |
| 48. | Talked to the client in a pleasant, friendly manner |  |  |  |  |  |  |
| 49. | Talked to the client with a harsh or negative tone |  |  |  |  |  |  |
| 50. | Looked at the client while talking to her |  |  |  |  |  |  |
| 51. | Looked at the client while the client was talking and actively listened |  |  |  |  |  |  |
| 52. | Praised client for what she is doing well |  |  |  |  |  |  |
| 53. | Asked the client if she had any other health-related questions or concerns |  |  |  |  |  |  |

1. Record the time the observation ended: ____ ____ : ____ ____
2. Observer’s comments:
