# Supplementary material for: Feasibility and acceptability of integrating a multicomponent breastfeeding promotion intervention into routine health services in private health facilities in Lagos State, Nigeria: A mixed methods process evaluation
Source: PLoS One. 2024 Apr 26;19(4):e0301695. doi: 10.1371/journal.pone.0301695 (PMC11051595; doi:10.1371/journal.pone.0301695)
Supplement: S3 File — (DOCX) [file pone.0301695.s003.docx]

**S3 File. Client Exit Interview Tool**

Thank you for agreeing to participate in this interview. I want to remind you that whatever you say during this interview will be kept confidential and will not be shared with the provider or anyone at this health facility.

**INFORMATION ABOUT INTERVIEW**

Date: Day ________ Month _______ Year ________

Name of interviewer: ________________________________________

Provider ID numbers: _____________________________

(RECORD PROVIDER IDs FROM OBSERVATION FORM)

Participant ID number: __________

Name of facility:

|  |  |
| --- | --- |
|  |  |
|  |  |
|  |  |
|  |  |

**OVERALL THOUGHTS ABOUT THIS VISIT**

1. In your own words, please tell me about what happened during your visit today. What happened first and then next and so on?

_____________________________________________________________________

_____________________________________________________________________

_____________________________________________________________________

_____________________________________________________________________

_____________________________________________________________________

| No. | QUESTIONS AND FILTERS | | CODING CATEGORIES |
| --- | --- | --- | --- |
| **ADVICE FOR BREASTFEEDING** | | | |
| 2. | *Is the client pregnant or postpartum?* | | Pregnant………………………>>2a  Postpartum……………………>>2b |
| 2a. | FOR PREGNANT WOMEN ONLY: Did any of the health care providers you talked to today ask you how you plan to feed your baby? | | Yes…………………………1  No………………………….0  Don’t remember…………..99 |
| 2b. | FOR POSTPARTUM MOTHERS ONLY: Did any of the health care providers you talked to today ask you how you are feeding your baby? | | Yes…………………………1  No………………………….0  Don’t remember…………..99 |
| 3. | Did any of the health care providers talk with you about breastfeeding today? | | Yes………………………….1  No……………………....0>>7  Don’t remember………99 >>7 |
| 4. | What advice or information about breastfeeding did any of the health care providers give you today?  DO NOT READ RESPONSES. RECORD YES/NO FOR EACH ITEM. | | |
|  | Advice | Response | Codes |
| a. | Breastfeed baby within 1 hour of birth |  | Yes…………………………..1  No…………………………...0  Don’t know………………….99 |
| b. | Feed baby colostrum |  |  |
| c. | Not giving baby water, glucose water, or a taste of food before starting to breastfeed the first time |  |  |
| d. | Exclusive breastfeeding to 6 months |  |  |
| e. | Not feeding water or other liquids before 6 months |  |  |
| f. | Breastfeed whenever the child wants |  |  |
| g. | Empty one breast before feeding on the other side |  |  |
| h. | How to position the baby for breastfeeding |  |  |
| i. | How to express and store breast milk |  |  |
| j. | Continue breastfeeding when mother is sick or pregnant |  |  |
| k. | Give no artificial teats, pacifiers or suckers to breastfeeding babies |  |  |
| l. | Other (specify) |  |  |
| 5. | How useful did you find the advice or information about breastfeeding you received today from any of the health care providers? | | Very useful………………….1  Somewhat useful……………2  Not useful at all……………..3  Don’t know………………..99  Refused…………………..999 |
| 6. | How confident are you that you can carry out the infant feeding recommendations suggested today by any of the health care providers? | | Very confident………………1  Somewhat confident…………2  Not confident at all..………....3  Don’t know………………..99  Refused…………………..999 |
| 7. | Did you tell any of the health care providers about any concerns or problems that you have related to breastfeeding? | | Yes…………………………...1  No……………………...0 >>12 |
| 8. | What types of problem(s) or concern(s) about breastfeeding did you bring up during the visit today?  DO NOT READ RESPONSES.  MULTIPLE RESPONSES POSSIBLE. | | Baby is not able to latch……...1  Mother has inverted nipples….2  Mother has sore nipples……...3  Mother feels she doesn’t have enough milk………………….4  Mother’s breasts are engorged.5  Mother has pain in breast…….6  Other (specify)……………...98 |
| 9. | Do you feel that your problems or concerns related to breastfeeding were adequately discussed and resolved? | | Yes……………………...1>>11  No……………………………0 |
| 10. | Why do you think the health care providers didn’t discuss the issue(s) you raised?  MULTIPLE RESPONSES POSSIBLE | | Not enough time……………..1>>12  He/she forgot………………...2>>12 He/she didn’t know answer….3>>12  He/she referred me to another  provider………………………4>>12  Other (specify)……………...98>>12  Don’t know………………..99>>12  Refused…………………..999>>12 |
| 11. | How satisfied are you with advice you received today related to your breastfeeding problem? | | Very satisfied………………..1  Somewhat satisfied...………..2  Not satisfied…………………3  Don’t know………………..99  Refused…………………..999 |
| **CLIENT SATISFACTION WITH TODAY’S VISIT** | | | |
| 12. | In general, which of the following statements best describes your opinion of the services you received at this health facility today? | | I am very satisfied with the services I received in the facility today…………………1  I am more or less satisfied with the services I received in the facility today………………..2  I am not satisfied with the services I received in the facility today………………...3  Don’t know………………..99  Refused…………………..999 |
| **OVERALL THOUGHTS ABOUT VISITS** | | | |
| 13. | When you come for regular ANC or postpartum visits to the clinic, what is the MOST important part of the visit to you?  DO NOT READ RESPONSES. | | Limited waiting time…………1  Have my questions answered...2  Make sure my baby is healthy.3  Make sure I am healthy………4  Learn new information………5  Solutions to health problems……………………..6  Prescriptions of medicine……7  Immunizations for my child….8  Other (specify)……………...98  Don’t know………………..99  Refused…………………..999 |
| 14. | What things do the health care providers do that you find useful or helpful?  DO NOT READ RESPONSES.  MULTIPLE RESPONSES POSSIBLE. | | Treat me kindly………………1  Share information with me…..2  Answer my questions………..3  Encourage me………………..4  Listen to my concerns……….5  Give me take-home materials..6  Refer me to additional resources…………………….7  Address my health problems..8  Other (specify)……………...98  Don’t know………………..99  Refused…………………..999 |
| 15. | What things do you wish the health care providers would do or help you with during visits?  DO NOT READ RESPONSES  MULTIPLE RESPONSES POSSIBLE. | | Treat me kindly………………1  Share information with me…..2  Answer my questions………..3  Encourage me………………..4  Listen to my concerns……….5  Give me take-home materials..6  Refer me to additional resources…………………….7  Address my health problems..8  Other (specify)……………...98  Don’t know………………..99  Refused…………………..999 |
| 16. | How could the ANC/delivery services be changed to best help women initiate breastfeeding within one hour of delivery?  DO NOT READ RESPONSES.  MULTIPLE RESPONSES POSSIBLE. | | Have more time with providers during visits to discuss breastfeeding questions and concerns during ANC visits….1  Receive more take-home educational materials at ANC visits…………………............2  Have more programming in the community where it’s easier to access in the prenatal period....3  Offer more communication through mobile phones in the prenatal period……….………4  Have more programming related to breastfeeding for other family members………..5  Have hospital policies that support early initiation of breastfeeding…………………6  Other (specify)……………...98  Don’t know………………..99  Refused…………………..999 |
| 17. | How could ANC/postpartum services be changed to best help women exclusively breastfeed for six months?  DO NOT READ RESPONSES.  MULTIPLE RESPONSES POSSIBLE. | | Have more time with providers during visits to discuss breastfeeding questions and concerns during visits………..1  Receive more take-home educational material at visits...2  Have more programming in the community where it’s easier to access…………………….......3  Offer more communication through mobile phones………4  Have more programming related to breastfeeding for other family members………..5  Other (specify)……………...98  Don’t know………………..99  Refused…………………..999  Not applicable (woman is pregnant)………………….9999 |
| **TRANSPORT TO HEALTH FACILITY** | | | |
| 18. | How much time did it take you to travel to this health facility today? | | Less than 30 minutes…..…….1  30-59 minutes………………..2  1-2 hours……………………..3  >2 hours………………4 |
| 19. | What mode of transportation did you use to get to the health facility? | | Private car or motorbike…….1  Private bicycle……………….2  Public transport………………3  Boat………………………….4  Foot…………………………..5  Other (specify)……………...98 |
