# Supplementary material for: Feasibility and acceptability of integrating a multicomponent breastfeeding promotion intervention into routine health services in private health facilities in Lagos State, Nigeria: A mixed methods process evaluation
Source: PLoS One. 2024 Apr 26;19(4):e0301695. doi: 10.1371/journal.pone.0301695 (PMC11051595; doi:10.1371/journal.pone.0301695)
Supplement: S4 File — (DOCX) [file pone.0301695.s004.docx]

*SUMMARIES AND QUOTES*

*IMPLEMENTATION*

- When sharing how breastfeeding counseling and support has changed at their facility since the program began, most replied that the activity is occurring more frequently than previously. Participants describe how the activity has “intensified” or how there is more emphasis on exclusive breastfeeding and/or early initiation.
  - *“We have been doing it before but when Alive and Thrive came it was like a full time. Alive and Thrive, it’s like they gave us a gateway, a proper way to do what we have been doing before. You know we are just teaching but they take it high, a higher level. They pushed us to a higher level of initiative of this moral and based on exclusive breastfeeding.”*
  - *“It has made [breastfeeding counseling] more frequent.”*
- Respondents indicated that the A&T program contributed to greater motivation among both clients and facilitators to achieve BF outcomes, and seeing the positive impact has resulted in client and provider satisfaction with the program. One provider mentioned that A&T support built her confidence and convinced her to take the importance of breastfeeding more seriously. Another provider mentioned how patients arrive at the facility early on group counseling days because they were eager to attend these health talks.
  - *“Most times when a patient says, ‘I don’t want to do exclusive’ you give up. When a patient says, ‘I need to buy formula’ you give up. When a patient says, ‘my mother said I should give water and I’m giving water’ you give up. But now, not anymore. We have to keep on following them, refreshing their mind on WhatsApp. We’re always refreshing their mind, sending out materials, you know, like encouraging them, announcing when they do well on the platform, everybody will be happy, congratulating them, ‘you did exclusive!’”*
  - *“I didn’t really take it this seriously, and the issue I had before was [I found it difficult] to gather mothers, then talk to them. But now that Alive and Thrive has [supported me], I was able to face them. You know, so many of those mothers you are seeing, they have mothers-in-law and some other people around them, they believe in those people. Sometimes one has to build confidence before talking to them. Alive and Thrive helped us to.”* Implementation Facilitators

The factors that helped participant integrate BF counseling and support into the services at the facility during this project

- The benefits of the materials were vast, serving as reminders for both providers and clients at the facility, or reinforcing messaging that occurs during other activities (e.g. group or individual counseling).
  - *“The fact that this materials are with them they see it all the time, it has become part of them so they don’t really need to struggle in any aspect of the counselling and guidance on the breastfeeding, you understand. When they look at them there on the wall, they are there. If you are moving around you just see them from that you pick a glance, I think it has help them a lot. So don’t struggle with most of the things like learner, it’s becoming part of them already”*
  - *“There are some people from the EHAI office that come and most times they give us materials that support us [on] everything that involves exclusive breastfeeding. They also gave us a guide of how to go about this while teaching the mothers, [the main points] that we are supposed to list out, that we’re not supposed to miss. So those materials that they gave us, actually helped us a great deal, in ensuring that we pass the information across to the mothers.”*
- Receiving support or encouragement – either from facility leadership, other providers, or A&T staff –strengthened relationships and facilitated program activities. Participants usually mentioned that this support/encouragement is a component of other interaction (e.g. training, facility visits) with A&T/EHAI staff. A&T/EHAI staff were supportive and encouraging in their interactions with facility staff as well as clients. Respondents also mentioned the importance of support from facility leadership, and support providers give their patients to achieve program goals. Reminders through this supportive interaction with A&T/EHAI staff, and from access to program materials, helped respondents implement program activities.
  - *“When the head is part of the whole process, it makes the whole thing fine, the MD is part of it, the GM is part of it, everybody is part of it, so, no need it will become very easy even the matron is part of it, so it become very easy for everybody to play along”*
  - *“They keep calling us, reminding us and they do come to our facility. Once I know that they are coming, I don’t want to fail on the assignment that they have already given to me. They come regularly to visit our facility to check my data, to check all what we are doing, to know what we are doing, whether we are doing what they ask us to do, it really encourages me.[…] All those factors really encourage me and help to discharge my duty appropriately”*
- Participants indicated different components of the training, including practical aspects of positioning the baby for breastfeeding, integrating interpersonal communication strategies, and receiving support/encouragement from trainers contributed to participants’ perception of training usefulness.
  - *“There was a day they brought this dummy something that looked like a baby. The way one of the doctors that came positioned the baby, it is as if I have never breastfed a baby before. Before, they just position the baby anyhow and you know some babies [would] be crying, they will think that the breastmilk is not sufficient for that child. Even when I was breastfeeding my child, I had cracked nipple, bruises; they taught us that it is bad positioning of baby that causes cracked nipples. By [the] time our mothers began to put their baby [the way we learned in the training], we did not hear any reports of, ‘my nipple is cracked.’ You know, she said that; “knowledge is power” is like I did not even know all this and I just thank God that they really helped to enlighten us-what they introduced has made our eyes to open to a lot of things I did not even know before.”*
  - *“During the training, we were able to learn about interpersonal relationships. I think that was the most essential thing there, because, though we have had the knowledge earlier now, at times we are not able to tell the mothers or have the confidence to talk to them or to correct them when they’re doing the wrong thing.”*
- Having an atmosphere that supports program activities throughout the facility was also an important facilitator. Providers appreciated being able to incorporate program messaging/activities when interacting with patients in different wards at different time periods (e.g. ANC, immunization), as it contributed to client acceptability. Generating a supportive atmosphere for clients – e.g. ensuring client privacy, integrating song/dance into activities – was helpful to motivate clients to participate.
  - *We asked everyone to come together as a team and work towards our goal. Alive & Thrive has really made every one of us come together as a team to push the [breastfeeding] policies forward.*
  - *“It is how we relate to them and how we bring it to them, before we start, we pray first, then we now sing some songs. When we hear those songs like that, it would make us happy. So, that is part of what makes them to relate with us”*
  - *“We start with the antenatal, and once we have prepared their mind, in the antenatal, we don’t really have challenges that much. We have already prepared their mind in the antenatal”*

How provision of BF counseling and support affects service delivery at the facility (staff time, staff availability)

- When asked whether the provision of BF counseling/support had any impact on staff time/availability, some respondents replied yes. They spoke of the increased workload being “cumbersome” and how they needed to make themselves available during non-working hours (e.g. responding to client queries via WhatsApp or making themselves available for training on their day off), that patient load has increased and had an impact on the amount of time it takes to do activities, and documentation being time consuming.
  - *“mothers will be asking questions in the midnight, calling you, most of them when they have emergencies maybe, most especially the first time mum, and the, first time mum, both pregnant and that have children, when they don’t know what to do when they encounter one difficulty or the other, they will have to call, not minding the time or send message either personal or to the group. Most times when they don’t get answers from the group, they tend to call personally.”* Implementation Challenges

The main barriers to integrating BF counseling and support into services at the facility during the project

- Participants cited challenges/barriers to implementing **phone/WhatsApp** activities. Clients were also at times frustrated when providers didn’t respond to questions immediately on the phone because they were busy with other responsibilities at the facility or they contacted providers at odd hours throughout the night.
  - *“For those of us that handle the WhatsApp group, when we see a mother waiting, we advise them that they can also private chat us or call us. But they’ll be calling us at odd hours. Some of them will be calling at 12 midnight, 1 am, 2 am to ask one or two questions and you’ll be wondering why they wouldn’t wait till morning. So that was just the challenge that me and the focal person faced. Because most of them ask questions at odd hours and before you’ll be able to answer them, ‘let’s meet in the morning’, and if you wake up from sleep you’ll answer them, some of them will get angry and exit from the group. That was just the only challenge”*
- Participants cited technical difficulties related to ensuring the phone was charged with data to conduct activities, that there was adequate network connectivity at the facility, and the memory capacity of the phone leading to them deleting names from the group.
  - *“I think it’s those women that don’t have much data or that don’t subscribe, so they feel like, whenever they enter WhatsApp, our messages will wipe all their data so, some of them just exit the group.”*
  - *“It has added to the workload. It’s like somebody was sweeping one room before, and then you now told the person to sweep 3 more rooms, without giving the person more hands to do the work”*
  - *“Time was actually a barrier. We have to make out time, and we have so many persons to attend to. I really, most time I have to try my best to squeeze out time to talk to them and when I have the time it really pays.”*

*TRAINING*

- Participants believed the primary benefits of the training are increased provider knowledge (most common response), that training contributed to providers taking breastfeeding more seriously, and training improved interactions with patients (which, in turn, resulted in improved child health outcomes). Lastly, another benefit is that providers can apply lessons learned in training not just to their patients, but also themselves and their wider communities.
- Owners/managers cited benefits of training mirrored those mentioned by health providers, and they noticed marked improvement in their staff interactions with patients as a result of the training.
  - *“I remember one of new employees we had […] they will just pick[ed] her to come and attend the training for those two days at the clinic and […] she wasn’t too good at it. After the training, one of the days after the lady delivered, the baby was moved to her ward. I remember that day, I didn’t have baby care, so, I joined her and I saw the way she helped the mother: she took her time to help the mother, to help the baby to latch, and it was quite encouraging to watch.”*
  - *“[…] Before I came to this facility—where there were no trainings – sometimes [the providers found] it difficult to do the counselling and the advice, discuss the advantages and all of that.[…] Here it [is] easy, [it] is not a problem because of training they’ve gotten. They will able to ensure that they teach the mothers on the positioning for proper breastfeeding. […] I think the [providers] here, they are better [than those at my previous facility] and I am sure it might be from the training they had. It is part of them now, doing the counselling and the teaching of the mother it just come[s] spontaneously not with any difficulty, it comes effortlessly”*
  - *“The benefit to us, to our facility, to my staff is that now we can comfortable talk to a mother about breastfeeding. Now they don’t see it as a waste of time. They invest their time on it because they are seeing results.”*
  - *“Wow! It has been, it has really been educating, it has been educating even to ourselves, and it has really helped us to be able to give ehm, counseling to all our clients as well. And the result, we’ve been really getting results from, through our babies.”*
  - *“For our staff, well, we still have some of them that are still active, in having babies, so they are able to pick one or two tips for themselves and also with that they are able to, able to advice their neighbors and people around them most especially advice the patients they come in contact with”*

*SUSTAINABILITY*

- Many of the participants indicated an interest in continuing the program, many stating their facility is already preparing to ensure activities continue after the program officially ends. Participants liked the program because of the benefits to their clients and believe it is important to continue activities after the program ends. Familiarity with the activities – either from before the program started or throughout the duration of the program – will facilitate continuation.
  - *“Our plan is that we will not change the program. We’ve been doing it since even before they introduced it, so we’ll continue”*
  - *“we’re planning to add the topic to our, our monthly office seminar. So that we will be refreshing our mind continuously on that.* *Because we’ve seen the result, we’ve seen that it’s very helpful. It made our work easier and it made our own understanding as professionals easier with education. We now know we can achieve a lot.”*
  - *“Well we have already started continuity, at least we have discussed with the MD about it. we’ve discussed with the MD, he approved it and we have created group for our mothers.* *So we have been adding them and educating them. That one has come to stay. There’s no how we can stop it.”*
- Owners/managers spoke of the importance of making these activities routine at their facilities, that the knowledge gained by facility staff will last past the end of the program, and that they believe in the benefit program activities have for their clients.
  - *“The learning was meant to be a learning that we take in and believe in it, it is not just to satisfy those that are coming to teach us, it is something that we are to know and to know how to keep implementing it, because, it is just a good thing to”*
