# Supplementary material for: Feasibility and acceptability of integrating a multicomponent breastfeeding promotion intervention into routine health services in private health facilities in Lagos State, Nigeria: A mixed methods process evaluation
Source: PLoS One. 2024 Apr 26;19(4):e0301695. doi: 10.1371/journal.pone.0301695 (PMC11051595; doi:10.1371/journal.pone.0301695)
Supplement: Attachment — Submitted filename: Response to Reviewers.docx [file pone.0301695.s007.docx]

July 30, 2023

Dr. Martin Wiredu Agyekum,

Guest Editor,

PLOS ONE

Dear Dr. Agyekum,

We are appreciative of the Editor and reviewers’ thorough review of our manuscript and insightful comments. We have provided our point-by-point responses to each of the comments below in blue text. We have also highlighted changes to the manuscript’s text using tracked changes.

1. Please ensure that your manuscript meets PLOS ONE's style requirements, including those for file naming. The PLOS ONE style templates can be found at

<https://journals.plos.org/plosone/s/file?id=wjVg/PLOSOne_formatting_sample_main_body.pdf> and

<https://journals.plos.org/plosone/s/file?id=ba62/PLOSOne_formatting_sample_title_authors_affiliations.pdf>.

RESPONSE

Thank you. This has been revised to ensure the manuscript meets PLOS ONE’s style requirements.

2. Please include a complete copy of PLOS’ questionnaire on inclusivity in global research in your revised manuscript. Our policy for research in this area aims to improve transparency in the reporting of research performed outside of researchers’ own country or community. The policy applies to researchers who have travelled to a different country to conduct research, research with Indigenous populations or their lands, and research on cultural artefacts. The questionnaire can also be requested at the journal’s discretion for any other submissions, even if these conditions are not met.  Please find more information on the policy and a link to download a blank copy of the questionnaire here: <https://journals.plos.org/plosone/s/best-practices-in-research-reporting>. Please upload a completed version of your questionnaire as Supporting Information when you resubmit your manuscript.

RESPONSE

A copy of the questionnaire has been uploaded as supporting information.

3. You indicated that you had ethical approval for your study. Please clarify whether minors (participants under the age of 18 years) were included in this study. If yes, in your Methods section, please ensure you have also stated whether you obtained consent from parents or guardians of the minors included in the study or whether the research ethics committee or IRB specifically waived the need for their consent.

RESPONSE

No minors were included in the study. All study participants were ≥ 18 years. We have included the following statement in the Methods section: “Women were eligible at enrollment to participate in the cohort if they were ≥ 18 years, in their third trimester of pregnancy, and were currently clients of a private health facility selected for the study.”

4. Thank you for stating the following in the Competing Interests section:

“SB is employed by Alive & Thrive and OA is employed by Equitable Health Access Initiative. SB was involved in managing the evaluation of the intervention and OA was involved in the design and implementation of the intervention, but not in data collection or analysis for this study. All other authors have no conflicts of interest.”

We note that one or more of the authors are employed by a commercial company: Equitable Health Access Initiative

a.         Please provide an amended Funding Statement declaring this commercial affiliation, as well as a statement regarding the Role of Funders in your study. If the funding organization did not play a role in the study design, data collection and analysis, decision to publish, or preparation of the manuscript and only provided financial support in the form of authors' salaries and/or research materials, please review your statements relating to the author contributions, and ensure you have specifically and accurately indicated the role(s) that these authors had in your study. You can update author roles in the Author Contributions section of the online submission form.

Please also include the following statement within your amended Funding Statement.

“The funder provided support in the form of salaries for authors [insert relevant initials], but did not have any additional role in the study design, data collection and analysis, decision to publish, or preparation of the manuscript. The specific roles of these authors are articulated in the ‘author contributions’ section.”

RESPONSE

The Funding Statement has been revised to reflect the recommendations of the editor.

If your commercial affiliation did play a role in your study, please state and explain this role within your updated Funding Statement.

RESPONSE

The Funding Statement has been revised to read: “This research was funded by The Alive & Thrive initiative, managed by FHI Solutions, and funded by the Bill & Melinda Gates Foundation (INV-029432 and OPP 1135932), Irish Aid, the Tanoto Foundation, UNICEF, and the World Bank. Alive & Thrive initiative provided support in the form of salary for Author SB but did not have any additional role in the design, data collection and analysis, decision to publish, or preparation of the manuscript. Equitable Health Access Initiative provided support in the form of salary for Author OA but did not have any additional role in the design, data collection and analysis, decision to publish, or preparation of the manuscript. The specific roles of these authors are articulated in the ‘author contributions’ section.”

b. Please also provide an updated Competing Interests Statement declaring this commercial affiliation along with any other relevant declarations relating to employment, consultancy, patents, products in development, or marketed products, etc.

RESPONSE

The Competing Interests Statement has been revised to read: “SB is employed by Alive & Thrive initiative. SB was involved in managing the evaluation of the intervention but was not involved in the design, data collection and analysis of the study. OA is employed by Equitable Health Access Initiative. OA was involved in the design and implementation of the intervention, but not in data collection or analysis for this study. OA’s affiliation with Equitable Health Access Initiative does not alter our adherence to PLOS ONE policies on sharing data and materials. All other authors have no conflicts of interest.”

Within your Competing Interests Statement, please confirm that this commercial affiliation does not alter your adherence to all PLOS ONE policies on sharing data and materials by including the following statement: "This does not alter our adherence to PLOS ONE policies on sharing data and materials.” (as detailed online in our guide for authors <http://journals.plos.org/plosone/s/competing-interests>). If this adherence statement is not accurate and there are restrictions on sharing of data and/or materials, please state these. Please note that we cannot proceed with consideration of your article until this information has been declared.

RESPONSE

The authors have confirmed this in the Competing Interests Statement.

Please include both an updated Funding Statement and Competing Interests Statement in your cover letter. We will change the online submission form on your behalf.

RESPONSE

The authors have included both the updated Funding Statement and the Competing Interests Statement in the cover letter.

5. We note that you have indicated that data from this study are available upon request. PLOS only allows data to be available upon request if there are legal or ethical restrictions on sharing data publicly. For more information on unacceptable data access restrictions, please see <http://journals.plos.org/plosone/s/data-availability#loc-unacceptable-data-access-restrictions>.

In your revised cover letter, please address the following prompts:

a) If there are ethical or legal restrictions on sharing a de-identified data set, please explain them in detail (e.g., data contain potentially sensitive information, data are owned by a third-party organization, etc.) and who has imposed them (e.g., an ethics committee). Please also provide contact information for a data access committee, ethics committee, or other institutional body to which data requests may be sent.

b) If there are no restrictions, please upload the minimal anonymized data set necessary to replicate your study findings as either Supporting Information files or to a stable, public repository and provide us with the relevant URLs, DOIs, or accession numbers. For a list of acceptable repositories, please see <http://journals.plos.org/plosone/s/data-availability#loc-recommended-repositories>.

We will update your Data Availability statement on your behalf to reflect the information you provide.

RESPONSE

The dataset needed to replicate our study’s findings have been uploaded as supporting information

6. Please review your reference list to ensure that it is complete and correct. If you have cited papers that have been retracted, please include the rationale for doing so in the manuscript text, or remove these references and replace them with relevant current references. Any changes to the reference list should be mentioned in the rebuttal letter that accompanies your revised manuscript. If you need to cite a retracted article, indicate the article’s retracted status in the References list and also include a citation and full reference for the retraction notice.

RESPONSE

The authors have reviewed the reference list to ensure completeness and correctness.

[Note: HTML markup is below. Please do not edit.]

Reviewers' comments:

Reviewer's Responses to Questions

**Comments to the Author**

1. Is the manuscript technically sound, and do the data support the conclusions?

The manuscript must describe a technically sound piece of scientific research with data that supports the conclusions. Experiments must have been conducted rigorously, with appropriate controls, replication, and sample sizes. The conclusions must be drawn appropriately based on the data presented.

Reviewer #1: Yes

Reviewer #2: Yes

2. Has the statistical analysis been performed appropriately and rigorously?

Reviewer #1: N/A

Reviewer #2: Yes

3. Have the authors made all data underlying the findings in their manuscript fully available?

The [PLOS Data policy](http://www.plosone.org/static/policies.action#sharing) requires authors to make all data underlying the findings described in their manuscript fully available without restriction, with rare exception (please refer to the Data Availability Statement in the manuscript PDF file). The data should be provided as part of the manuscript or its supporting information, or deposited to a public repository. For example, in addition to summary statistics, the data points behind means, medians and variance measures should be available. If there are restrictions on publicly sharing data—e.g. participant privacy or use of data from a third party—those must be specified.

Reviewer #1: Yes

Reviewer #2: Yes

4. Is the manuscript presented in an intelligible fashion and written in standard English?

PLOS ONE does not copyedit accepted manuscripts, so the language in submitted articles must be clear, correct, and unambiguous. Any typographical or grammatical errors should be corrected at revision, so please note any specific errors here.

Reviewer #1: Yes

Reviewer #2: Yes

5. Review Comments to the Author

Please use the space provided to explain your answers to the questions above. You may also include additional comments for the author, including concerns about dual publication, research ethics, or publication ethics. (Please upload your review as an attachment if it exceeds 20,000 characters)

Reviewer #1: The paper presents the findings of a robust intervention.
The introduction is adequate.
The methods did not mention why the authors used the specific number of women that were interviewed for each component (structured observation/exit interviews)

RESPONSE

We thank the review for the comments. We have included the following description of the study’s processes used in selecting the specific number of women who were interviewed for each component of the study in the Methods Section:

“The sample sizes of 20 for the IDIs and 179 for the structured observations and exit interviews were selected to allow for representation of all facilities and comparisons between intervention and comparison facilities.” Lines 193-195

There are no concerns regarding research ethics.
The line numbering in the document is not continuous (page 20 ended at 354, page 21 started at 332)

RESPONSE

Thank you for calling this to our attention. The line numbers have been updated to ensure they are continuous.

In the discussion:
It will be helpful if Line 352 - 354 on page 23 in the discussion can throw more light on line 352 - 354 on page 20 (result), Explain why in spite of the counseling "fewer mothers in the intervention (77%) compared with comparison health facilities (87%) reported feeling very confident they could carry out the advice they had received".

RESPONSE

During the 6 weeks postpartum visits exit interviews, more mothers from the intervention health facilities (77%) reported receiving counseling on not feeding infants water or other liquids to infants before 6 months, compared to mothers from the comparison health facilities (73%). The authors believe that the health providers’ emphasis on mothers refraining from giving water and other liquids to babies < 6 months, a predominant cultural practice in Nigeria, contributed to fewer mothers from the intervention health facilities (77%) feeling very confident they could carry out the advice they had received compared to the comparison health facilities (87%).

Looking at all the things that were put in place in this study to achieve success, did any of the respondents give information regarding sustainability? If yes, can it be included?

RESPONSE

Yes. Sustainability of the intervention was discussed in the IDIs. We have added the following text to the Results and Discussion Sections:

RESULTS

“Many of the health providers reported that breastfeeding counseling and support for pregnant women and lactating mothers would continue even after the intervention period ended:

“*Well, we have already started [thinking about] continuity. At least we have discussed with the MD[Managing Director] about it and he has approved it and we have created a [Whatsapp] group for our mothers. So, we have been adding them and educating them. That one has come to stay. There’s no way we can stop it.” (Female health provider, 2 years at health facility).*”” Lines 314-320

DISCUSSION

“Many of the health providers reported plans to systematize providing breastfeeding counseling and support to pregnant women and lactating mothers.” Lines 405-406

Reviewer #2: The article adds to the need for critical interventions related to breastfeeding support and promotion and is therefore a much needed study at this time, particularly in a busy metropolitan city like Lagos. Please note that: (1) sub Saharan Africa as a region should begin with caps..eg. Sub NOT sub. See indications throughout the work and revise accordingly.

RESPONSE

Thank you for calling this to our attention. The texts in the manuscript have been revised to reflect this recommendation.

(2). Details regarding the frequency of text messages including number of messages /texts daily/weekly should be mentioned within the section where it is described to provide better reading and easy replicability

RESPONSE

We thank the reviewer for this comment. We have revised the methods to include more details: “The intervention also included an mHealth component in which pregnant women and lactating mothers received breastfeeding messages through daily text messages/SMS sent in bulk; and could participate in breastfeeding support groups on WhatsApp, led by an assigned ‘Breastfeeding Champion.’” Lines 157-160

6. PLOS authors have the option to publish the peer review history of their article ([what does this mean?](https://journals.plos.org/plosone/s/editorial-and-peer-review-process#loc-peer-review-history)). If published, this will include your full peer review and any attached files.


If you choose “no”, your identity will remain anonymous but your review may still be made public.


**Do you want your identity to be public for this peer review?** For information about this choice, including consent withdrawal, please see our [Privacy Policy](https://www.plos.org/privacy-policy).

Reviewer #1: No

Reviewer #2: **Yes:** Ajike Saratu O

[NOTE: If reviewer comments were submitted as an attachment file, they will be attached to this email and accessible via the submission site. Please log into your account, locate the manuscript record, and check for the action link "View Attachments". If this link does not appear, there are no attachment files.]

While revising your submission, please upload your figure files to the Preflight Analysis and Conversion Engine (PACE) digital diagnostic tool, <https://pacev2.apexcovantage.com/>. PACE helps ensure that figures meet PLOS requirements. To use PACE, you must first register as a user. Registration is free. Then, login and navigate to the UPLOAD tab, where you will find detailed instructions on how to use the tool. If you encounter any issues or have any questions when using PACE, please email PLOS at [figures@plos.org](mailto:figures@plos.org). Please note that Supporting Information files do not need this step.

Sincerely,

Diana Allotey

Department of Nutrition,

University of North Carolina at Chapel Hill
